# Perceived psychosocial stress and gestational weight gain among women with gestational diabetes

**DOI:** 10.1371/journal.pone.0174290

**Published:** 2017-03-28

**Authors:** Ai Kubo, Assiamira Ferrara, Susan D. Brown, Samantha F. Ehrlich, Ai-Lin Tsai, Charles P. Quesenberry, Yvonne Crites, Monique M. Hedderson

**Affiliations:** 1 Kaiser Permanente Division of Research, Oakland, California, United States of America; 2 Department of Public Health, University of Tennessee Knoxville, Knoxville, Tennessee, United States of America; 3 Department of Obstetrics and Gynecology, Kaiser Permanente Medical Center, Santa Clara, California, United States of America; University of Tennessee Health Science Center, UNITED STATES

## Abstract

Growing evidence links perceived stress—a potentially modifiable psychosocial risk factor—with health behaviors and obesity. Yet little is known about the relationship between stress during pregnancy and gestational weight gain, particularly among women with pregnancy complications. We conducted a cross-sectional analysis to examine associations between psychosocial stress during pregnancy and gestational weight gain among women with gestational diabetes. We used baseline data from the Gestational Diabetes’s Effects on Moms (GEM) study: 1,353 women with gestational diabetes who delivered a term singleton within Kaiser Permanente Northern California were included. Perceived stress near the time of gestational diabetes diagnosis was measured using the validated Perceived Stress Scale (PSS10). Gestational weight gain was categorized according to the 2009 Institute of Medicine recommendations. Binomial regression analyses adjusted for gestational age and maternal age at the time of gestational diabetes diagnosis, and race/ethnicity and estimated rate ratios (RR) and their 95% confidence interval (CI). Among women with a normal pregravid Body Mass Index (BMI 18.5–24.9 kg/m^2^), there was a significant association between high (Q4) PSS score and risk of both exceeding and gaining below the Institute of Medicine recommendations compared to those with lower stress (Q1) [adjusted RR = 2.16 95% CI 1.45–3.21; RR = 1.39 95% CI 1.01–1.91, respectively.] Among women with pregravid overweight/obesity (BMI≥25 kg/m^2^), there was no association. Although the temporal relationship could not be established from this study, there may be a complex interplay between psychosocial stress and gestational weight gain among women with gestational diabetes. Further studies examining stress earlier in pregnancy, risk of developing gestational diabetes and excess/inadequate gestational weight gain are warranted to clarify these complex relationships.

## Introduction

Excess or inadequate gestational weight gain (GWG) has serious health consequences for pregnant women and their offspring. Women who gain excessive weight during pregnancy are more likely to have a caesarean section delivery;[1] pre-eclampsia;[1] and excessive weight retention after delivery.[2] Inadequate gestational gain also has adverse consequences with birth outcomes such as increased risk of preterm birth and small for gestational age.[3] Furthermore, gestational diabetes (GDM), a common pregnancy complication has been found to increase the risk of perinatal complications,[4] most notably fetal overgrowth (i.e. macrosomia)[5, 6] and the associated birth injuries due to the large size of the fetus, progression to type 2 diabetes in the mothers, and obesity and type 2 diabetes in the offspring.[7–9] The potentially synergistic adverse effects of excess or inadequate GWG and GDM makes understanding factors associated with optimal GWG in this high risk population particularly important.

Existing studies suggest that maternal pregnancy stress may influence perinatal outcomes including GWG by disrupting the hypothalamic pituitary axis (HPA), resulting in elevation of cortisol levels and promotion of intake of nutrient-dense “comfort” food and central fat distribution.[10–12] Despite the biological plausibility of this framework, previous observational studies have failed to demonstrate an association between psychosocial stress during pregnancy and the risk of excess or inadequate GWG among women with uncomplicated pregnancies.[13] However, no previous study has examined this association among women with gestational diabetes (GDM). Psychosocial stress is a potentially modifiable, yet understudied potential risk factor that is prevalent among pregnant women,[14, 15] particularly those diagnosed with pregnancy complications such as GDM.[16, 17]

We conducted a cross-sectional analysis to explore whether there is an association between perceived stress during pregnancy and GWG, classified in accordance with the 2009 IOM recommendations for GWG,[18] among women diagnosed with GDM, using a large multiethnic cohort delivering at Kaiser Permanente Northern California (KPNC).

## Materials and methods

**Fig 1:**
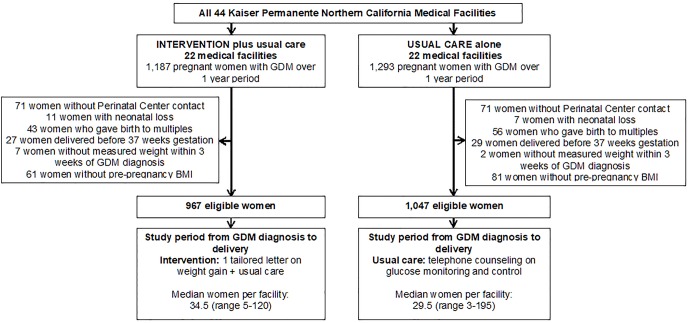
Overview of the pregnancy component of the GEM cluster randomized trial.

This study is a secondary cross-sectional analysis of baseline data collected for the “Gestational Diabetes’s Effects on Moms (GEM)” trial at KPNC (Fig 1). Details of the GEM trial are described elsewhere.[19] Briefly, GEM was a cluster randomized clinical trial of the comparative effectiveness of diabetes prevention strategies for women with GDM. A telephonic lifestyle intervention was implemented after the delivery. The study was approved by the Kaiser Permanente institutional review board. The human subjects committee of the Kaiser Foundation Research Institute waived the requirement for individual informed consent for the intervention component, given the pragmatic, cluster randomized design.[19] All intervention and data assessment materials for the GEM study have been approved by the human subjects committee of the Kaiser Foundation Research Institute; the trial is also registered at clinicaltrials.gov (NCT01344278).

From March 2011 to March 2012, all women with a diagnosis of GDM according to the Carpenter and Coustan criteria, as recommended by ACOG during the study period,[20] who were 18 years of age or older were identified in KPNC’s electronic health record (EHR). Women were contacted soon after the diagnosis of GDM and invited to participate in the baseline GEM survey, in English or Spanish. Among all 2,280 eligible women in the 44 medical facilities who were sent study surveys,[19] 1,783 (78.2%) completed the baseline questionnaire. Survey responders did not differ from non-responders except for being less likely to be of non-Hispanic white origin.[19]

### Measurement of maternal stress during pregnancy

All study subjects were asked to complete at baseline: a mail- or web-based survey of lifestyle, demographic information, and physical and psychological health. Women completed the baseline survey after the GDM diagnosis (median gestational weeks 32.1 (interquartile range (IQR) 29.0–34.6)). We used the Perceived Stress Scale (PSS10),[21] a well-validated scale with Cronbach’s alpha of 0.87 within our study sample, to obtain information on perceived stress during the month before the survey. PSS10 has been found to correlate well with stressful life events and depressive symptoms and predicted health outcomes (physical symptomatology and visits to a health care center) better than life event measures.[21] PSS10 has been validated and widely used in a variety of studies involving pregnant women.[8, 22] In our study, PSS10 was scored according to established protocol.[21, 23] The sum of all 10 questions was calculated for the final PSS10 score. Stress was categorized into quartiles, with the lowest quartile as the referent.

### Outcome measurements

Pregravid BMI was calculated from a clinic-measured pregravid weight measured within 12 months of conception obtained through the electronic health record (EHR; 86.0%), weight ascertained at the first prenatal clinic visit before 10 weeks’ gestation (10.5%, from the EHR), or self-reported pregravid weight on the GEM pregnancy survey (3.5%). To validate this method of estimating pregravid weight, we compared the self-reported pregravid weight and first pregnancy weight to the measured weight among women who had both information: the Pearson correlations were 0.97 between measured weight and self-reported pregravid weight (mean difference (SD) 0.7 kg (9.4); 453 women), and 0.99 between measured weight and first measured weight during pregnancy (mean difference (SD) -2.2 kg (6.7); 943 women). Information on measured height was obtained from the electronic health record. Pregravid BMI was calculated as the pregravid weight (kg) divided by the height (m) squared. Last measured weight in pregnancy (within 2 weeks of delivery), as measured by KPNC clinical staff was abstracted from the EHR.

Participants' total gestational weight gain was calculated as the last measured weight in pregnancy (within 2 weeks of delivery) minus pregravid weight. The IOM provides pregravid BMI-specific recommended ranges for rate of GWG in the 2nd and 3rd trimester and a recommendation for absolute weight gain in the first trimester that allows for accounting for the gestational week at delivery.[18] We categorized women’s total weight gain as below, within or above the IOM recommended weight gain range for her BMI and gestational week at delivery using the IOM’s rate of weight gain recommendations.

### Covariates

We used participant surveys and KPNC electronic databases to collect data on age (continuous), race (white, non-white), household income (less than $50,000, $50,000–100,000, more than $100,000), maternal education (high school graduate or less; some college, 2 year college, technical degree; 4-year college graduate or postgraduate degree), energy intake over the past 3 months (kcal/day, continuous, measured with the Block Food Frequency questionnaire[24]), percent of calories from fat over the past 3 months (continuous, measured with the Block Food Frequency questionnaire[24]), and physical activity over the past 3 months (total MET hours/week, continuous, measured with the Pregnancy Physical Activity Questionnaire[25]).

### Statistical analysis

First, univariate analyses were conducted to describe the baseline characteristics of the women included in the analysis. We compared proportions and means of demographic, health and lifestyle information, stratified by perceived stress level. To determine if the differences are statistically significant, we conducted Chi-square test for categorical variables, ANOVA for normally distributed continuous variables, and Kruskal-Wallis tests for non-normally distributed continuous variables. We then conducted log binomial regression analyses to assess the associations between the quartiles of perceived stress and adequacy of GWG. Given randomization at the medical center level in the parent study (n = 44 facilities), estimation of regression parameters was by generalized estimation equations (GEE) with clustering by center. Outcomes were total GWG exceeding or not meeting the IOM recommendations.

For multivariate analysis, the model was adjusted for gestational age, maternal age at the time of GDM diagnosis, and race/ethnicity. Other covariates did not change the effect estimate substantially (>10%) therefore were not included in the final model. We assessed effect modification by including a cross-product term (stress*pregravid BMI). Because there was significant interaction (p<0.01), the results are grouped by pregravid BMI (normal; overweight; and obese). To assess the potential mediating role of diet and physical activity in the association between stress and excess or inadequate GWG, we also ran models including energy intake (kcal/day), % calorie from fat, and physical activity (total MET hours/week). For these analyses, we excluded those with extreme caloric intake report (<600 or >4000 kcal/day; n = 62) and those with extreme physical activity (total hours of activity >20 or hours of moderate intensity activity per day >9; n = 103). Tests for interaction with race/ethnicity, education or income were not statistically significant. All analyses were performed using SAS statistical software (Cary, NC). The study and analyses were approved by KPNC institutional review board.

## Results

Among the 1,783 women who completed the baseline survey, we excluded those who did not fill in the stress questionnaire (n = 408) or were missing data for GWG (n = 1). We also excluded women whose pregravid BMI was below 18.5 (n = 21) since this group was too small to include in the sub-group analysis. Our final sample included 1,353 women with GDM who delivered a term singleton (≥ 37 weeks’ gestation). Those who were included in the analyses were not substantially different from those who were not included in the analyses with regard to demographic characteristics or obesity status. The PSS10 was completed at a median of 32.1 weeks gestation (IQR 29.0–34.6). Overall, the cohort had a median total GWG of 9.6 kg (IQR 6.2–13.6); 28.3% of the cohort gained below the IOM recommendation, 34.6% met the recommendation, and 37.1% exceeded the recommendation.

### Baseline characteristics by stress levels

Table 1 presents the baseline demographic and lifestyle characteristics, and obesity status of study participants, stratified by the quartile categories of PSS10 score. The ranges for each quartile were 6–10, 13–15, 16–19, and 21–25 for Q1, Q2, Q3, and Q4, respectively. There were no significant differences in demographic characteristics across the quartiles of stress level, except that women with higher education were more likely to be in lower stress categories (Q1 or Q2) than their less educated counterparts (p = 0.02). Total caloric intake, percent of calories from fat and physical activity levels were similar between the groups.

**Table 1:** Demographic characteristics by perceived stress scale (PSS) score; Gestational Diabetes Effects’ on Mom (GEM) study.

Stress level (PSS10 score)	Q1	Q2	Q3	Q4	p-value
PSS10 range	6–10	13–15	16–19	21–25	
N	368	335	305	345	
Characteristic	N (%)/Mean(SD)	N (%)/Mean(SD)	N (%)/Mean(SD)	N (%)/Mean(SD)	
**Age at baseline, years**					
18–29	107 (29)	86 (26)	78 (26)	113 (33)	0.12
30–34	138 (38)	135 (40)	112 (37)	137 (40)	
35–46	123 (33)	114 (34)	115 (38)	95 (28)	
**Race/ethnicity**					
Black/African American	12 (3)	10 (3)	11 (4)	16 (5)	0.75
Caucasian	95 (26)	79 (24)	81 (27)	85 (25)	
Hispanic	79 (21)	66 (20)	52 (17)	66 (19)	
Asians	141 (38)	149 (44)	129 (42)	133 (39)	
Multiracial or others	41 (11)	31 (9)	32 (10)	45 (13)	
**Parity**					
Nulliparous	176 (48)	138 (41)	118 (39)	147 (43)	0.10
Multiparous	192 (52)	197 (59)	187 (61)	198 (57)	
**Education**					
High school graduate or less	54 (15)	57 (17)	40 (13)	65 (19)	**0.02**
Some college, 2 year college, technical degree	117 (32)	88 (26)	113 (37)	122 (35)	
A 4-year college graduate or postgraduate degree	194 (53)	190 (57)	151 (50)	157 (46)	
**Annual household income**					
Less than $50,000	98 (27)	83 (25)	78 (26)	118 (34)	
$50,000-$99,999	132 (36)	124 (37)	112 (37)	111 (32)	
$100,000 and greater	122 (33)	106 (32)	96 (31)	94 (27)	
**Pregravid BMI,kg/m2**					0.86
18.5–24.9	122 (33)	114 (34)	102 (33)	108 (31)	
25.0–29.9	107 (29)	108 (32)	87 (29)	106 (31)	
30.0+	139 (38)	113 (34)	116 (38)	131 (38)	
**Energy intake, kcal/day**	1708 (640)	1781 (1095)	1795 (803)	1870 (876)	0.10
**% of Kcal from fat**	41.9 (7.0)	42.7 (6.8)	42.4 (7.3)	41.7 (7.1)	0.24
**Physical activity, MET minutes/week [median (interquartile range)]**	1500.0 (810.0–2475.0)	1447.5 (780.0–2445.0)	1485.0 (840.0–2520.0)	1350.0 (690.0–2302.5)	0.48

BMI = Body Mass Index

MET = Metabolic Equivalent

### GWG by stress levels and pregravid obesity

Table 2 shows proportions of women who had inadequate, adequate and excessive GWG categorized by stress levels and pregravid BMI. Generally, higher proportion of women in the highest quartile of stress score tended to exceed the IOM recommendation for total GWG compared to those in the lowest stress category, but the associations were significant only among those with pregravid BMI<25 (p = 0.02).

**Table 2:** Total GWG by stress level and pregravid BMI; Gestational Diabetes Effects’ on Mom (GEM) study.

Total Gestational Weight Gain		Q1	Q2	Q3	Q4	p-value
N		N (%)	N (%)	N (%)	N (%)	
		368	335	305	345	
**PSS10 Score (range)**		**8 (6–10)**	**14 (13–15)**	**18 (16–19)**	**24 (21–25)**	
**Pregravid BMI**	**GWG**					
**18.5–24.9**	Inadequate	35 (28.7)	37 (32.5)	29 (28.4)	38 (35.2)	**0.0158**
	Adequate	65 (53.3)	41 (36.0)	48 (47.1)	34 (31.5)	
	Excessive	22 (18.0)	36 (31.6)	25 (24.5)	36 (33.3)	
**25–29.9**	Inadequate	20 (18.7)	30 (27.8)	23 (26.4)	17 (16.0)	0.1915
	Adequate	41 (38.3)	35 (32.4)	31 (35.6)	33 (31.1)	
	Excessive	46 (43.0)	43 (39.8)	33 (37.9)	56 (52.8)	
**≥30**	Inadequate	49 (35.3)	37 (32.7)	34 (29.3)	36 (27.5)	0.7801
	Adequate	34 (24.5)	32 (28.3)	34 (29.3)	34 (26.0)	
	Excessive	56 (40.3)	44 (38.9)	48 (41.4)	61 (46.6)	

### Association between perceived stress and excess GWG by pregravid BMI

Table 3 presents the association between PSS10 score and the risk of exceeding the IOM recommendation for total GWG categorized by pregravid BMI. Among women with a normal pregravid BMI, a high level (Q4) of stress was significantly associated with over twice the risk of exceeding IOM recommendations compared with women with low stress (Q1) [Rate Ratio (RR) = 2.16, 95% CI 1.45–3.21], after adjusting for maternal age and race/ethnicity. There was a significant test for trend across the stress categories for excessive GWG (p = 0.02). High stress level was also associated with the risk of inadequate GWG [RR = 1.39 95%CI (1.01–1.91)], though test for trend was not statistically significant. On the other hand, there was no association between stress level and the risk of excess or inadequate GWG among women who were overweight or obese prior to their pregnancy.

**Table 3:** Adjusted rate ratios from log multinomial regression estimating the association between perceived stress level and exceeding or being below the IOM recommendation for total GWG; Gestational Diabetes Effects’ on Mom study.

	Stress Level		Q1		Q2		Q3		Q4	P for trend
Pregravid BMI	GWG	N	RR (95%CI)	N	RR (95%CI)	N	RR (95%CI)	N	RR (95%CI)	
**18.5–24.9**	Inadequate	35	1.0 (ref)	37	1.24 (0.88–1.73)	29	1.02 (0.72–1.44)	38	1.39 (1.01–1.91)	0.15
	Adequate	65		41		48		34		
	Excessive	22	1.0 (ref)	36	1.84 (1.23–2.75)	25	1.35 (0.86–2.11)	36	2.16 (1.45–3.21)	0.002
**25–29.9**	Inadequate	20	1.0 (ref)	30	1.37 (0.80–2.37)	23	1.25 (0.70–2.23)	17	1.02 (0.54–1.96)	0.99
	Adequate	41		35		31		33		
	Excessive	46	1.0 (ref)	43	1.03 (0.75–1.41)	33	1.01 (0.72–1.43)	56	1.17 (0.91–1.49)	0.18
**≥30**	Inadequate	49	1.0 (ref)	37	0.91 (0.70–1.17)	34	0.85 (0.62–1.16)	36	0.88 (0.66–1.16)	0.29
	Adequate	34		32		34		34		
	Excessive	56	1.0 (ref)	44	0.92 (0.70–1.22)	48	0.95 (0.74–1.21)	61	1.05 (0.83–1.32)	0.66

Adjusted for maternal age and race/ethnicity

### Test for mediation by diet and physical activity

These aforementioned associations were not attenuated by adjustment for diet or physical activity. For instance, among women with normal pregravid BMI, comparing upper quartile of stress score to the referent, models that included total caloric intake [RR = 2.18, 95% CI 1.47–3.23], percent of calories from fat [RR = 2.25, 95% CI 1.20–2.74] or total caloric intake and physical activity [RR = 2.27, 95% CI 1.50–3.45] did not differ from the model that did not include these lifestyle variables [RR = 2.16, 95% CI 1.45–3.21]. Adjustment for any combinations of these variables resulted in similar effect estimates (data not shown).

A sensitivity analysis restricted to women with a measured pregravid BMI found similar results (data not shown). We conducted a sensitivity analysis to determine whether the effect of stress on GWG was driven by maternal or fetal weight gain by calculating total maternal GWG after subtracting infant birth weight. We found that compared to women with a low PSS stress score (Q1), having a high PSS score stress (Q4) increased the risk of being in the highest quartile of maternal GWG compared to being in the lowest quartile of maternal GWG [RR 1.20 95% CI: 1.04–1.44]. This suggests that maternal stress is associated with increased maternal pregnancy weight gain, independent of fetal growth.

## Discussion

Our study suggests that there may be an association between high perceived stress during pregnancy and excess or inadequate GWG among women diagnosed with GDM who had a healthy BMI prior to the index pregnancy. The association between high stress and excess GWG persisted when we examined maternal pregnancy weight gain (defined as total pregnancy weight gain minus infant birthweight) suggesting maternal stress was impacting maternal weight independent of fetal growth. This study, although not designed to investigate temporal relationship, suggests that there may be complex interplay between perceived stress and gestational weight gain among women with GDM.

Reports from the White House Task Force on Childhood Obesity[26] and the U.S Surgeon General[27] recommend promoting effective perinatal interventions and research on health factors affecting weight gain during pregnancy in diverse populations in an effort to interrupt the intergenerational cycle of obesity. Furthermore, the 2009 IOM report highlights the need to understand the role of psychosocial factors in optimizing GWG.[18] Women with pregnancy complications such as GDM are more likely to have a higher level of psychological stress compared to women with a healthy pregnancy.[16, 17, 28] However, the association between stress and GWG among women with GDM has been understudied.

There is some evidence supporting a relationship between psychosocial stress and/or depression and weight gain from the overall population of pregnant women. A study by Picone et al. examined psychological stress as a factor in GWG and pregnancy outcome in a small study and found a correlation between higher stress scores and lower GWG, independent of nutrient or caloric intake.[29] The authors concluded that perhaps stress did not directly affect food intake, but rather that it may have impacted the utilization of calories and nutrients from the foods consumed to support pregnancy. The 2009 IOM report, “Weight Gain During Pregnancy: Reexamining the Guidelines,” reviewed the existing literature on depression and gestational weight gain and concluded most studies suggested that both low and high GWG may be a sign of depression during pregnancy.[30] These data from general pregnant populations suggest that different effects of stress and depression on GWG can occur depending on how an individual responds to stress. The IOM report highlighted the need for more research on the role of stress in gestational weight gain.[8, 30] The association between psychosocial stress and risk of exceeding or gaining below the GWG recommendations was observed only among women with a normal pregravid BMI. For the present study, we speculate that the effect modification might have been observed because overweight or obese women are already at higher risk of exceeding the recommended GWG compared to women with a normal pregravid BMI;[31, 32] therefore, perceived stress had no additional effect on excess weight gain.

Further, some women’s stress levels might have been affected by the diagnosis of GDM. Women with the diagnosis of GDM are often put on a strict diet to optimize weight gain, therefore some women may have overcompensated the dietary regimen resulting in less overall weight gain. Among women with higher BMI, there may be many genetic, psychological, and/or environmental risk factors explaining why they exceed the recommended GWG, masking the effect of psychosocial stress on GWG and resulting in the observed effect modification by pregravid BMI.

There have been numerous behavioral intervention studies conducted in an effort to optimize GWG and reduce the risk of maternal pregnancy complications, though the results are mixed.[33, 34] In a review by Skouteris, et al., the authors suggested a need for the consideration of psychological factors relevant to pregnancy, in addition to health behaviors, including healthy eating and increased physical activity.[34] Pregnancy is a time of considerable psychological and physiological stress for many women.[14, 15] The presence of a diagnosed pregnancy complication such as GDM further escalates the level of stress and anxiety,[16, 17, 28] making stress reduction a potential candidate for future prevention strategies.

There are several limitations in this study. First, this study only included women with GDM and their stress level was assessed a few weeks after their GDM diagnosis. Since we did not assess their perceived stress prior to the GDM diagnosis, we are unable to assess whether stress increased the risk of GDM among these women or GDM diagnosis affected the level of stress, and how that impacted gestational weight gain up to the time of diagnosis. A previous study by Silveira et al. reported that an *increase* in stress level between early to mid-pregnancy substantially increased the odds of developing GDM among Hispanic women, compared to those with no change or a reduction in stress.[35] Although we were unable to assess the temporal relationship between stress during the early stage of pregnancy and the risk of developing GDM or excess/inadequate GWG, our study extended previous findings by suggesting that there may be associations between stress around the time of GDM diagnosis and increased risk of excess or inadequate total GWG. Second, although we did not observe mediating role of diet and physical activity, it is possible that the measures were not accurate, resulting in lack of observation. More rigorous method of diet and exercise measures are needed to better understand the role of these variables in the association between stress and weight gain during pregnancy. Third, we were unable to examine whether maternal stress had an effect on glycemic control and whether that may have impacted GWG; future studies are needed to clarify the inter-relationship between stress, glycemic control and GWG. However, we tried to assess confounding by including as many potential factors that were available as possible in the model, including income and education; none of these variables explained the observed associations (data not shown). Fourth, although we did not observe interaction between education, race/ethnicity and income, it is possible that the study did not have enough power to detect the effect modification. Lastly, there may be different types of stress (e.g., HPA of SAM responses) that we were unable to distinguish in the present study because PSS10 only measures perceived stress. If more evidence is found on the association between stress and GWG, further study examining how different types of stress affect weight gain may be warranted.

In conclusion, psychosocial stress may be an independent risk factor for gaining outside of the IOM’s recommended GWG range among women with GDM who had a normal BMI prior to the pregnancy. Future longitudinal studies of pregnant women to evaluate the interplay of stress, measured at multiple times during pregnancy, weight gain, and changes in blood glucose levels are warranted. If an association exists, future intervention studies may include stress reduction programs in conjunction with modifying lifestyle to improve the effectiveness of interventions to optimize GWG, thereby preventing future intergenerational transmission of obesity and diabetes.
